# Data, Disasters, and Space-Time Entanglements

**DOI:** 10.1007/s13753-021-00333-x

**Published:** 2021-02-15

**Authors:** Eija Meriläinen, Mirka Koro

**Affiliations:** 1 Institute for Risk and Disaster Reduction and Institute for Global Health, University College London, London, WC1E 6BT UK; 2 Department of Management and Organisation, Centre for Corporate Responsibility, Hanken School of Economics, 00101 Helsinki, Finland; 3 Mary Lou Fulton Teachers College, Arizona State University, Tempe, AZ 85281 USA

**Keywords:** Chile, Creata, Data, Disaster studies, Qualitative disaster research, Space-time

## Abstract

Disasters connected to natural hazards can at the same time be unfolding events, as well as structural phenomena with unequal disaster risk constructed over an extended timespan. Hence, in disaster studies, temporality and spatiality are central, yet often implicit, concepts employed to make sense of the disaster phenomena. In this article we explicitly focus on temporality and spatiality within qualitative disaster studies, particularly those containing ethnographic elements. We use Doreen Massey’s idea of space-time trajectories to analyze and illustrate how in qualitative disaster studies the trajectories of the disaster, research participants, and the researcher entangle in diverse ways. The focus is on how temporality and spatiality are present in the construction of data. The article is mainly conceptual, with illustrations drawn from empirical fieldwork on Valparaíso fire of 2014 in Chile. We interrogate how researchers’ sensitivity to temporality and spatiality challenges the conventional notions and practices of “data” in qualitative disaster studies. The focus in this article is on disaster studies, but it also offers methodological insights to other social sciences that strive to conduct research in the era of “Anthropocene,” with all its shifts and changes, the root causes of which have built over a long time.

## Introduction

Disasters appear to be becoming the status quo: hazards such as hurricanes have been intensifying (Mousavi et al. 2011) and the day-to-day conditions of most living entities are getting more dire (Cardinale et al. 2012; IPBES 2019), to name only a few of the disastrous shifts in the planet’s condition. The geological era that the planet currently experiences has been titled the “Anthropocene”^1^ to draw attention to the human-driven changes in the functioning of the Earth system (Steffen et al. 2007). Scientists caution that unless human societies adopt dramatic changes in how they and their institutions function, the Earth system might pass tipping points beyond which the future is bleak and uncertain (Biermann et al. 2012). The Anthropocene thesis, Clark (2014, p. 19) argues, can be interpreted as the “positing of a disaster to end all disasters.” This seemingly contrasts the figures showing that prior to the COVID-19 pandemic the loss of life resulting from reported, large-scale natural hazards had been on the decline globally, a trend that is arguably due to improved living standards and better governance of disasters (CRED and UNISDR 2019). However, these figures do not fully capture the everyday disasters and the environmental devastation that is brewing. The degradation of environmental conditions, coupled with stark societal inequalities, demands disaster governance that digs beyond the localized disaster risk, and delves into the long-term root causes across all scales (Tierney 2012; Kelman et al. 2015; Mehrabi et al. 2019).

Social scientists could strive to tackle the challenges of the Anthropocene both through questioning the established and problematic ontological assumptions embedded in bodies of knowledge, as well as through supporting political mobilization (Lövbrand et al. 2015). For instance, the ways in which nature is typically evoked as an object standing apart from society represent a problematic trace of positivism embedded in the broad set of social sciences (Lövbrand et al. 2015). To advance knowledge production within disaster studies also calls for shifting and diversifying established research practices, epistemologies, and methodologies (Fiddian-Qasmiyeh 2019; Gaillard 2019). Although the potential shifts could involve many things, our focus here is on the treatment of temporality and spatiality^2^ in conducting disaster research.

Disasters are studied within a variety of literature streams, which range from physiological accounts of a hazard on a limited timespan, to sociopolitical analyses of the long-term buildup and effects of disasters. Disaster studies deal with the temporal and spatial unfolding of complex and devastating phenomena in a range of ways, at best making visible how the structures of the past weave into the shifts and shocks of the present to lay out possible futures, for better or for worse (Pelling and Dill 2010; Barclay et al. 2019). This article focuses on temporality and spatiality in qualitative disaster studies, a body of knowledge that can: (1) benefit from a more explicit discussion of temporality and spatiality in conducting qualitative research; as well as (2) illustrate some issues of conducting qualitative research in what appears to be the turmoil of the Anthropocene, with the potential of informing social sciences more broadly.

In the context of conducting empirical disaster research, we continue to promote the quest for temporal and spatial sensitivity, articulated, for instance, by thinkers engaging with postmodernity, postmodernism, and post-qualitative movements within qualitative research (Strohmayer and Hannah 1992; Koro-Ljungberg et al. 2017). Our illustrations draw from fieldwork in Chile and we focus on disasters entangled with “natural” hazards, such as earthquakes or forest fires. These types of disasters are, firstly, structural phenomena where disaster risk follows unequal sociospatial configurations, often constructed over extensive periods of time, that define the people and places that are likely to face devastation following a hazard (see Oliver-Smith 1979). Secondly, disasters connected to “natural” hazards are also unfolding events that have impacts on various temporal (see Bankoff 2004) and spatial scales. The relevant temporal scales range from the seconds that an earthquake might last to the hundreds of years that it might take for the associated disaster risks to build up. The relevant spatial scales, meanwhile, range from individuals having to leave their homes to seek shelter to the involvement of regional, national, and transnational actors in disaster governance.

In making sense of disasters, qualitative researchers ebb and flow between the particular (for example, realities in the field) and the general (realms of theorizing). By embarking on a quest for temporal and spatial sensitivity, we do not mean to fixate only upon the particular(s), and slip into the realm of unqualified relativism and antifoundationalism for which postmodern thought has been (not always justly) criticized (Duncan 1996). Rather, we are interested in how the relationship between the particular empirical insights and the realm of theorizing is created in disaster studies, in terms of temporality and spatiality. One major concern in this regard is that explanations emanating from local concerns and conceptualizations of the dominant places in the “West” or “global North” (Massey 2004), are used, also within disaster studies, to frame phenomena elsewhere (Bankoff 2019).

In this article, the aim is to interrogate how researchers’ sensitivity to temporality and spatiality challenges conventional notions and practices of “data” in qualitative disaster studies. Regarding the politics of knowledge production, the questions of “data” continue to be relatively unexplored and ignored (Koro-Ljungberg 2013; Koro-Ljungberg et al. 2017). We are particularly interested in the temporalities and spatialities embedded in the creation of data within disaster studies. In order to keep us (the authors) and you (the readers) on our toes about our assumptions about what data are and highlight their constructed nature, we refer here to the concept of “creata,^3^” rather than data (Bendix-Petersen 2013). Furthermore, we explore how space-time trajectories (Massey 2005)—of the researcher, research participants, and disasters—entangle in conducting qualitative disaster studies to create what is labelled as “data” or “creata.”

This article continues with a review of spatiality and temporality in qualitative research (Sect. 2). This is followed by a section on ideas about and practices of constructing and analyzing “data” that also introduces the concept of “creata.” The fourth section then explores creata in conducting qualitative disaster studies research on and between entangling space-time trajectories, and draws illustrations from the qualitative research with ethnographic elements conducted by the first author (Meriläinen 2020). The diverse elements of the article are brought together in the fifth section, which is followed by a brief concluding section.

## Spatiality and Temporality in Qualitative Research

According to Giddens (1990), premodern cultures’ conceptions of time tended to be entwined with place, binding “when” and “where.” As the use of the mechanical clock spread, time became to be measured increasingly uniformly. The uniformity of time and, relatedly, of social organization travelled alongside the expansion of modernity that saw the ideas of space and time grow increasingly separated. Emptying of time was a precondition to “emptying of space,” by which Giddens (1990) means the intellectual separation of local place and universal space. Modern understandings came to problematically view “place” as an apportioning of space, merely its fraction (Casey 1996). Colonialism—with Europeans “discovering” regions considered far-off from their origins—contributed further to the emptying of space through making spatial units substitutable and hiding the traces of privileging a distinct vantage-point (Giddens 1990). The emptying of space, as well as the disconnection between the universal and the particular, have further deepened under capitalism (Harvey 2000; Smith 2010).

Modernist perspectives on time and space have been critiqued on many fronts, yet their legacy continues to be embedded in various social science disciplines. Disaster studies are not an exception. In order to discuss spatiality and temporality together in a meaningful way, in this article we have opted to build on Doreen Massey’s thought (2005). To counter the tendency of many dominant spatiotemporal conceptualizations to sever the connection between the particular and the universal, Massey (2005) argues against evoking the binary between place and space—and space and time. Within human geography, Massey can be credited with time-sensitive approaches that link past, present, and future (Anderson 2010). While other thinkers, such as Lefebvre (2013), have had similar aspirations, we find Massey’s (2005) vocabulary and thinking particularly appropriate for the aim of this article.

Massey (2005) recognizes how political movements and conservative communities leverage the idea of place to signal locality, meaningfulness, and the concrete realm of the everyday, while space is treated as global, meaningless, and abstractly hollow. She rejects this binary approach, and her space-place continuum consists of a living constellation of trajectories. Material place plays an important role in providing the chance for shared space, a general condition and opportunity for being together. In Massey’s words:

Place, in other words does—as many argue—change us, not through some visceral belonging (some barely changing rootedness, as so many would have it) but through the practicing of place, the negotiation of intersecting trajectories; place as an arena where negotiation is forced upon us. (Massey 2005, p. 154)

Massey (2005) illustrates how space turns to time, and geography to history, in the context of the development of countries. Here her thinking echoes with Latin American post-development authors in that she argues against seeing all locations as part of a single trajectory of development where some places are thought to be behind others (Massey 2005; Gudynas 2013). Instead Massey (2005, p. 5) tries to awaken an imagining of “contemporaneous heterogeneities of space.”

We build on Massey’s (2005) conceptualizations of spatiality and temporality in thinking about data within qualitative research, and qualitative disaster studies in particular. While qualitative and quantitative research are at times put in tension with each other, they are complementary approaches in which qualitative research might fare particularly well in mapping out the development of social phenomena in real time (Silverman 2016). Qualitative research encompasses a broad range of theories and approaches across diverse fields of social research. Because diverse fields come with their own (dominant) epistemologies and methodologies, there is no one way of conducting qualitative research.

Although much of the discussion in this article remains relevant for other types of qualitative disaster studies as well, our focus is on qualitative disaster studies that involve ethnographic elements. Ethnography is a “people-focused emic research which makes use of data collection methods such as participation, observation, and interview, and which unfolds by the way of description and interpretive conceptualization” (Vannini 2015, p. 318). While anthropologists may have developed ethnography into a scholarly form of art, various other fields have incorporated ethnographic elements into their work. For instance, in geography Hitchings and Latham (2019) observe that authors deploying the concept “ethnographic” tend to communicate that their research strives to place the respondents’ interviews amidst their lived social action. A key concept in ethnography is the idea of “the field.” In ethnography, fieldwork implies a researcher staying for an extended period in a place, engaged in interaction with research participants “on their home ground” (Van Maanen 2011, p. 2).

A stream of literature on multi-sited ethnography has also debated the methodological questions related to conducting research between various space-time trajectories—though not necessarily expressed in those terms. While some multi-sited ethnographic work emphasizes multiple localities that research is conducted in and about (Falzon 2009), others emphasize ethnography as mobile, with researcher taking “unexpected trajectories” and weaving into ethnography spatial thinking (Marcus 1995, p. 96). However, as “multi-sited ethnography necessarily impl[ies] some form of (geographical) spatial de-centeredness” (Falzon 2009, p. 2), it is not the most appropriate label for our endeavor here, as we discuss disasters rooted, however fleetingly, in the place and time of a hazard unfolding. We believe that Massey’s thinking on trajectories provides a helpful tool for thinking about spatiality and temporality in relation to disaster studies. We will return to this discussion with illustrations in Sect. 4, when we consider how data are collected—or “creata” are constructed—in disaster studies.

## Notions and Practices of Data/Creata in Qualitative Research

In qualitative research, data often continue to be seen as “evidence” that a researcher “collects” from reality (Bogdan and Biklen 2006). This kind of conceptualization of data may be in line with positivist research approaches, but as perspectives on ways to conduct qualitative research have diversified and developed, understandings of data have remained problematically stagnant in comparison (Koro-Ljungberg 2008, 2010; St. Pierre 2013). Data appear to be a “heritage-concept” passed from a generation of social scientists to the next without being challenged (Koro-Ljungberg 2013). Simple, easily identifiable, countable, stable data could have existed in past methodological discourses and practices, but these conceptions of data go poorly together with many contemporary theoretical perspectives (Koro-Ljungberg et al. 2018). In the diverse contemporary field of qualitative research, data should no longer be treated as an independent object to be encountered and analyzed: it can be selected, produced, constructed (Brinkmann 2014), and much more.

In traditional qualitative research, data are often interpreted to come to life somewhere between the experiences of the research participants and the documentation of those experiences (Savin-Baden and Howell Major 2013). For example, the way in which data tend to be labelled and stored as a specific object, recorded under date, place, and “respondent” names attests to this (Patton 2002). Instead of treating data as a predictable and easily organized object or providing simple definitional answers, many qualitive researchers have become more hesitant and unsure about what “data” are (Denzin 2013; St. Pierre 2013; Koro-Ljungberg et al. 2017). Data can be seen as a process that is constructed within, through, and across diverse cultural events (see Marshall and Rossman 2015). “Data” as a process is likely to contain the seeds and fragments of its own analysis.

Bendix-Petersen (2013) adopted the concept “creata” in order to highlight the interconnectedness of cultural contexts, and the diverse processes of data creation and analysis. Data are not raw, available, and relevant nuggets of reality that a researcher happens to come across, but examples, fragments, analytics, and relations put together and initiated by research participants and shaped by their understanding of the phenomenon, lives, and realities studied. In conducting qualitative research, the conventional understanding of data as an “object” is particularly problematic for two reasons: it falsely assumes the separation (1) of the data and the researcher who is “collecting” and interpreting it, as well as (2) that of data and analysis (Bendix-Petersen 2013).

The first problem with conventional ideas about data is that they are assumed to be separate from the researchers and their positionality (Bendix-Petersen 2013). Data—or “creata” —get their life from an embodied and positioned being and while positionality can show itself in the conventional forms of data—such as in field notes—it can also be carried in the body as affects that guide the form of textual outputs (Bendix-Petersen 2013). Positionality typically refers to a researcher’s position in and across societies, and their personal experiences and beliefs that might shape the knowledge production they undertake (Rose 1997; Berger 2015). Different positionalities between researchers of the same or related disciplines can influence greatly the interpretation of the same data set (Dean et al. 2017), but the differences in findings can be even starker when the data are both “collected” and “analyzed” by differently positioned researchers using different theoretical lenses. As an example, studies on the same Mexican village of Tepotzlan, conducted only some 10 years apart, came to contradictory findings due to different theoretical framings that were entwined with the selection, coverage, and organization of data (Lee and Newby 1983; Morison 1989).

The positionality of researchers and their access to the field and different cultural realities vary. A researcher’s positionality shapes research and creata in three ways: (1) through access to the field (for example, the nature of interactions within communities); (2) through the nature of the relationship between the researcher and the researched (for example, what is shared); and (3) through the ways in which the researcher theoretically, philosophically, and materially constructs the world (Berger 2015). While being an “insider” to a phenomenon might add nuance to what a researcher captures of a cultural and geographical context, it may come at the peril of projecting one’s own experiences over and upon those of other research participants (Berger 2015). Meanwhile, particularly when conducting research with/on/among vulnerable or marginalized people, a researcher’s positionality and power position are unlikely to align entirely with those of the other research participants (Mayorga-Gallo and Hordge-Freeman 2017). While some researchers engage in activism alongside the places and people with whom they are studying (Nagar 2014; Cordeiro et al. 2017), not many researchers are likely to share the concerns and space-time trajectories of the marginalized populations extensively. Hence what creata comes to being—and what does not—relates to the ways in which the space-time trajectories of the researcher and the phenomenon do and can entangle.

A second problem with the conventional “object” conceptualization of data concerns how data and analysis are assumed and portrayed as separate and separable (Bendix-Petersen 2013). In conventional social science texts the authors first present the data and then proceed to analyze it, as if the “data” would not already contain seeds and fragments of analysis regarding the social phenomenon studied (Bendix-Petersen 2013). The issue is that while analysis may be a theoretical reading of data (Kvale and Brinkmann 2009), a researcher is likely to construct data with theoretical preconceptions in mind (Jones III and Gomes 2010). In that sense all qualitative research is abductive, with the reality of the studied phenomenon faced “in the field” entangling with the theoretical understanding of the phenomenon. After construction of data, a researcher is often driven to seek closure, stabilize meanings, or present truths (Kaufmann 2011). Bendix-Petersen (2013) rejects the dualism between construction of data and its analysis, arguing them to be entangled.

Data are necessarily contaminated by theory (Jones III and Gomes 2010), but the researcher is not the only source of theorizing. Instead, theorizing and analysis seep into data also from other research participants, their context, and the materiality of the worlds studied. While within academic institutions there continues to be a strong bias in what kind of positionalities are present in conducting research and theorizing on behalf of the rest (Peake 2011; Zenker and Kumoll 2011), theorizing itself is not the privileged domain of the Occidental researcher. The capacity to think theoretically is a trait of all humans, beyond specific and privileged geographic and time coordinates (Mignolo 2000). Hence “creata” constructed is not the reality of specific time-space coordinates bottled up by a researcher and preserved for later theoretical scrutiny. Rather, it is a process of construction and creation among research participants, ebbing and flowing between specific moments of space-time and theorizing that reaches, cumbersomely and on occasion dysfunctionally, towards the universal. Although the academic researcher and academic theory may hold more power over the creata process than other research participants, it remains relational and entangled with the positionalities and theorizing of research participants. Research participants do not simply hand over information about their realities naively, but rather they are “politically motivated actors” who care about the representation of phenomenon at hand (Alvesson 2011, p. 29). The following section discusses the whens and wheres of creata and their construction in the context of disaster studies.

## Creata and Entangling Space-Time Trajectories in Disaster Studies

In this section we explore how creata within qualitative disaster research are constructed,on and between entangling space-time trajectories. We enter into the discussion on temporality and spatiality through a specific focus on construction of creata. In doing so, we (1) draw from contemporary methodological discussions; (2) build on Massey’s (2005) thinking about space-time trajectories; and (3) explore the construction of creata within disaster studies through illustrations from the qualitative research with ethnographic elements conducted by the first author (Meriläinen 2020). Before moving on to the illustrations, this section positions our work into the broader scholarship of temporality and spatiality within disaster studies.

In the English language, the concepts “hazard” and “disaster” carry separate meanings: an earthquake or a flood might be considered a hazard, but disaster is defined as the devastating aftermath facing the people, society, and nature (Kelman 2018). The currently dominant paradigm in disaster studies (Gaillard 2019) emphasizes that while hazards may be natural, disasters are not. Who is affected, and in which manner, is more a matter of how societies are organized, than it is of the type of a hazard (O’Keefe et al. 1976; Chmutina and von Meding 2019).

We align with the “vulnerability paradigm” in arguing that a disaster is woven into the everyday and its resource and power inequalities (see Gaillard 2019). Seen from this perspective, disasters are constructed over extensive periods of time, and disaster risk reflects the inequalities of long-term, sociospatial configurations (Oliver-Smith 1979; Nygren 2018). The lives and existences of marginalized people are also likely to be exposed to various entangled threats (Kelman et al. 2015). Those deemed “vulnerable” might even be less concerned about extraordinary shocks than everyday risks, such as those related to livelihood security and physical infrastructure (Cannon 2008; Ruszczyk 2018).

While “vulnerability” can draw attention to long-term structural buildup of disasters, the approach has its Western biases (Bankoff 2019). Furthermore, over the past decades, it has been sidelined by “resilience” approaches that render vulnerability individual and voluntary, rather than structural and relational (Bankoff 2019). In order to draw attention to the relationality of vulnerability, Collins (2010), for instance, has suggested discussing the dual processes of marginalization/facilitation that construct unequal disaster risk within societies, as well as across them.

Beyond the lack of relationality, vulnerability perspectives can also be criticized for insufficiently giving agency to time (Bankoff 2004). Hazards and disasters may be characterized based on how the phenomenon unfolds temporally, for instance sudden-onset and slow-onset phenomena (Van Wassenhove 2006). However, following the vulnerability paradigm, all disasters should be considered “slow-onset” (Lewis 1988) and human-made. As such, they should be studied and theorized with an attunement to everyday inequalities and power relations, rather than with a fixation on the exceptionality of hazards as temporally and spatially limited events (Hewitt 1983; Mika 2019). Drawing from the historian’s perspective, Bankoff (2004) argues that in addition to highlighting disaster as a process, there might be a further need in the social sciences to explore the meaning of disasters as events, which we in this article refer to as the “eventness” of disasters.

While we agree that disasters should primarily be framed as slow-onset phenomena, we believe that eventness is present (Bankoff 2004), particularly in the methodology of disaster studies. When conducting empirical research on disasters, the time and place of a hazard event may form a starting point for the inquiry. There is a tendency in disaster studies to conduct inductive research (Lindell 2013) in the aftermath of a hazard (Norris 2006). “Outsider” disaster researchers are likely to gravitate towards large-scale disasters to collect “perishable” data, potentially posing a strain on disaster-affected communities and sidelining local researchers (Gaillard and Gomes 2015).

Beyond the methodology of disaster studies, various event-focused temporalities and spatialities seep into disaster research through the positionalities and conceptualizations of researchers and research participants. While time and space of a disaster are made sense of in diverse ways between actors, a hazard is likely to form a fixed point on space-time trajectories. For instance, dominant political actors and formal disaster governance actors are likely to emphasize the phases of disaster governance, such as mitigation, preparedness, response, and recovery. This phase-based approach centers the disaster event and reinforces the linearity of time where before, during, and after disaster follow one another (Aijazi 2014). In the response phase, Zebrowski (2019) argues that political actors strive to suppress events and their “disruptive” time quickly, aiming to restore the linear time of the everyday. Disaster recovery perspectives, similarly, tend to emphasize the role of institutional actors in addressing the political threat of disasters as events, even if an emphasis on resuming everyday life and rehumanizing the self might be more relevant for disaster-affected people (Aijazi 2014). Preparedness, meanwhile, uses future events as the justification for “action in the here and now” to mitigate the disruption caused by the threat to a life that is valued (Anderson 2010, p. 778). As such, preparedness may not so much highlight the linearity of time, but it does center the event.

Examples of spatial analyses of disasters abound. Although in some models space appears as a modernist container, qualitative disaster research in particular can challenge this perspective. Oliver-Smith (2010), discussing forced displacement and inspired by social mobilization following the Yungay earthquake of 1970, argues against seeing displacement as a natural way of the modern world. Rather, sense of place is central to being, and “uprooting people from the environments in which the vast majority of their meaningful activities have taken place and on which their understanding of life is based” is likely to be violent (Oliver-Smith 2010, p. 11). Despite the importance of place, disaster studies should not interrogate it merely as singular and bounded. For instance, a focus on diaspora groups can challenge the spatial boundedness of a disaster (Sewordor et al. 2019), while exploring the evolution of post-disaster neighborhoods can illustrate how disasters are rarely experienced by a singular and insulated “place” sharing a single temporal trajectory (Barrios 2014).

In the following sections, we present three illustrations from the qualitative research with ethnographic elements of one of the authors, illustrating how creata are negotiated in the intersection of the space-time trajectories of the disaster, the affected people, and the researcher. From a disciplinary standpoint, the work draws mostly from the broad umbrella of disaster studies and human geography. Here Massey’s (2005) thinking provides a useful analytical framework that brings together temporality and spatiality without reinforcing the problematic dichotomies of space/time and place/space. The illustrations are drawn from a set of data/creata that consists of some 100 days of observation, 15 semistructured formal interviews, many informal discussions, and seven field visits documented in written notes, photographs, and video clips (Meriläinen 2020). Additionally, secondary creata consist of various forms of documentation, from reports to online content (Meriläinen 2020). The illustrations center around a fire that could be construed as a sudden-onset “natural” hazard, in that the fire unfolded within a short time span, and without a directly verified human source.

### Space-Time Trajectories Entangling with a Disaster in Place

The fires that erupted in the hills of Valparaíso, a Chilean port city, on 12 April 2014 were later labelled as the *Gran incendio de Valparaíso*, the Great Fire of Valparaíso. The title of the events highlights the phenomenon as a single incident where a hazard (fire) ravaged certain hills of the city for a few days. The fire and the following damage unfolded unexpectedly within a short time window, even if the conditions that facilitated the spreading of the fire were built over time. The background conditions ranged from lightweight flammable construction material to unclear fire-fighting responsibilities in the wildland-urban interface where the fires started (Reszka and Fuentes 2014).

Although the hazard can be seen as spatially and temporally confined, the way the disastrous aftermath unfolded for the people differed. Some were directly affected (for example, losing lives, livelihoods, and people close to them) while others felt an indirect impact (for instance, supporting the affected population in diverse ways; being traumatized by the media portrayals). Because of the disaster, the ways in which different peoples’ and places’ space-time trajectories became entangled were diverse. To a great extent, the entwining of space-time trajectories followed the lines of structural inequalities embedded in the society. For example, the marginalized informal and low-income settlements were affected the most directly and for longer periods of time, while other parts of the city’s places and people only witnessed the fire from a distance, as ash in the sky and news in the media.

For the people who lost their homes in the fire, reconstructing or reclaiming housing was one of the most urgent fire-related concerns at the time of the study, one year after the fire. While some residents had stayed in the affected area—or were preparing to return—others had either left the hills or been moved out of the way of activities undertaken in the name of hazard preparedness and development. For many residents, the disaster appeared as continuous and ongoing.

**Fig. 1:**
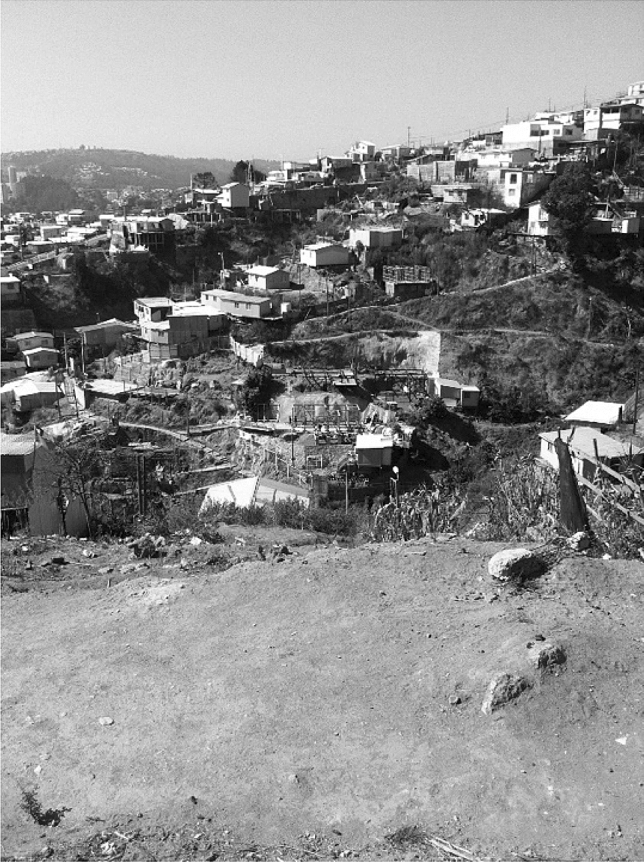
Revisiting hills of Valparaíso, Chile, one year after the fire, with María who coordinated students’ aid efforts following the fire. Photograph by Meriläinen, 2015

Soon after the fire vast number of individuals and organizations stepped in to help the affected people and animals, such as street dogs. One year on from the fire, most people helping in the aftermath of the fire had already moved on. For example, student volunteers from local universities had removed burnt items and provided other forms of support in the immediate aftermath. While a few students were still involved in 2015, most were gone in the days and weeks following the fire. The first author visited the affected hills with María,^4^ who had been coordinating the students’ aid efforts. The rebuilt homes and newly green bushes signaled to an outsider that the events that started unfolding in April 2014 had already passed (Fig. 1).

### A Space-Time Trajectory of a Research Participant Entangling with Several Disasters

While the Valparaíso fire of 2014 was labelled as “great” due to the widespread devastation in its wake, this was not the first fire in Valparaíso—nor was it certainly the first disaster to take place on the landmass currently hosting the Chilean nation-state. Chile rests on a tectonically active region and not only does it experience frequent and strong earthquakes (for example, the Valdivia earthquake in 1960), over the course of the year 2015 alone, several other hazards, such as mudslides, floods, and volcano eruptions, unfolded into disasters across the country that lengthwise ranges over 4000 km.

**Fig. 2:**
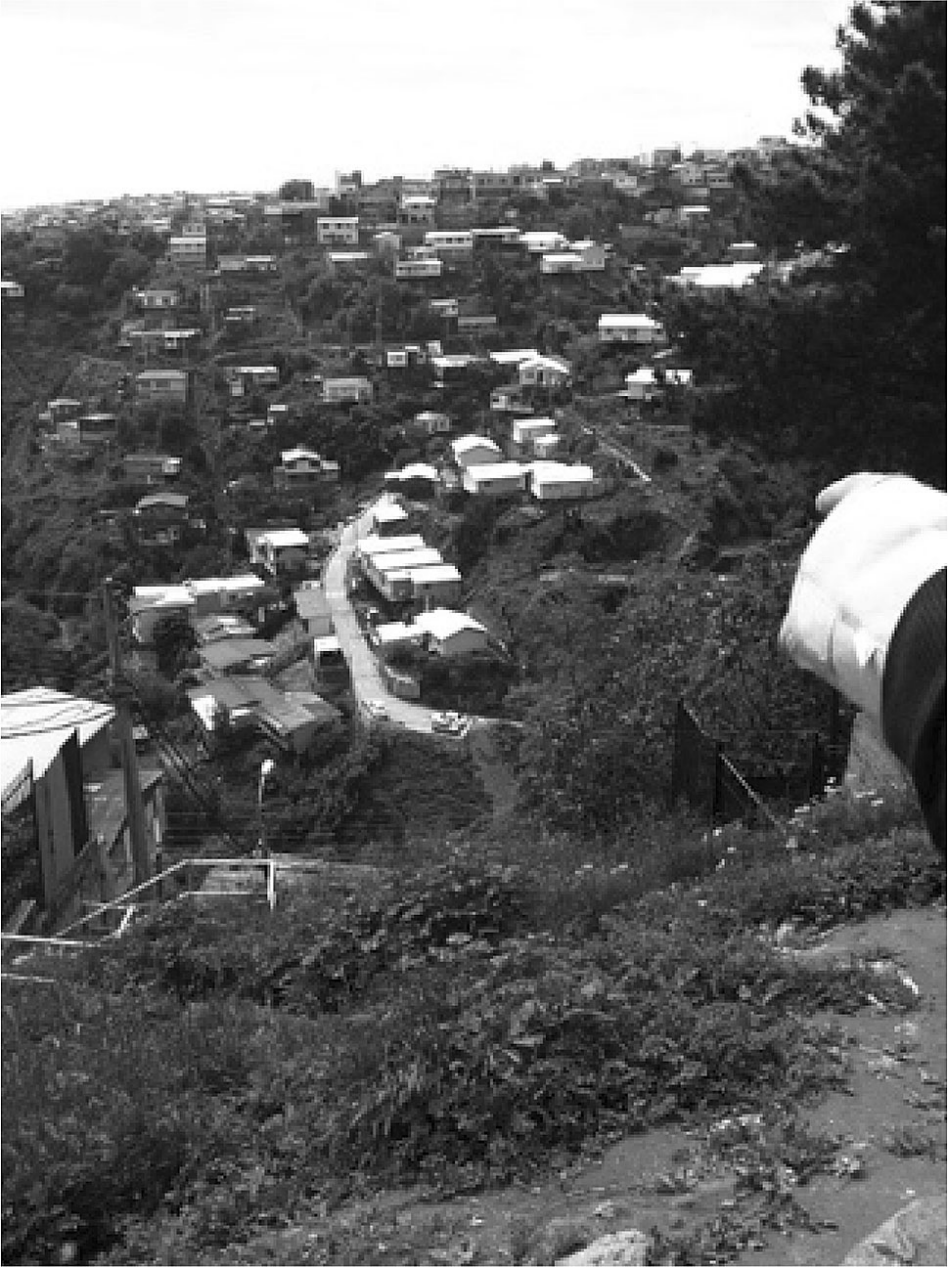
Hills of Valparaíso, Chile. Marco pointing to where the fire of 2013 unfolded near his home. Photograph by Meriläinen, 2015

Disasters are not necessarily singular and isolated events for the people facing them either. For example, the first author met with a teacher called Marco to discuss the Valparaíso fire of 2014 in a cafeteria, away from the affected hills. In making sense of the progression of 2014 events, Marco returned to two other disasters that had affected him personally. First, he discussed the 2010 earthquake and tsunami that occurred in the region where he had been working. Second, he brought up the fires of 2013 that took place in the hills where he currently lived. In the middle of the interview he suggested changing our meeting location in order to witness the place where the 2013 events had unfolded two years previous (Fig. 2).

While disasters may seem like unlikely events, some people and places are likely to be exposed to multiple threats (Kelman et al. 2015) that might in meaningful and painful ways entangle with their lives and space-time trajectories. Furthermore, there are organizations (such as nongovernmental aid organizations) and other actors (such as volunteers) that might not themselves be encountered by a disaster, but who seek to encounter them.

### A Researcher Entangling with a Disaster

The first author had planned to go to Chile to study the aftermath of the 2010 earthquake, but, eight months prior to her arrival, the Valparaíso fire erupted close to the university she was about to visit, changing her plans. She followed online media accounts regarding the fire while still in her “home country,” *Suomi* (Finland in English)*,* but the actual field research began in 2015 when she moved to the Valparaíso region. It was then that her space-time trajectory started entangling with those of the people and places shaped by the disaster. Through interviews and field visits, documented in audio recordings, scribbled notes, and photographs, she strove to build a bridge between the events that happened on 12 April 2014 and the processes that were still unfolding. It was in creata that the first author aimed to capture the present and past that then had been.

During the year 2015 the first author remained mainly an outsider compared to those space-time trajectories that had become most entangled with the disaster. She lived relatively comfortably and was a visiting researcher at a local private university, which meant that she did not share any material consequences of the disaster—even if the imagination of the events haunted her in the documented and the undocumented creata. Although her semiperipheral identity would diverge from the “Western” or “European” norm, the positionality that she found herself in, particularly in Chile, was just that. Many people related to the first author by assuming she was a U.S. American or a German, relating anecdotes to those places and people (Meriläinen 2018). The positionalities experienced or projected shaped what creata were constructed.

**Fig. 3:**
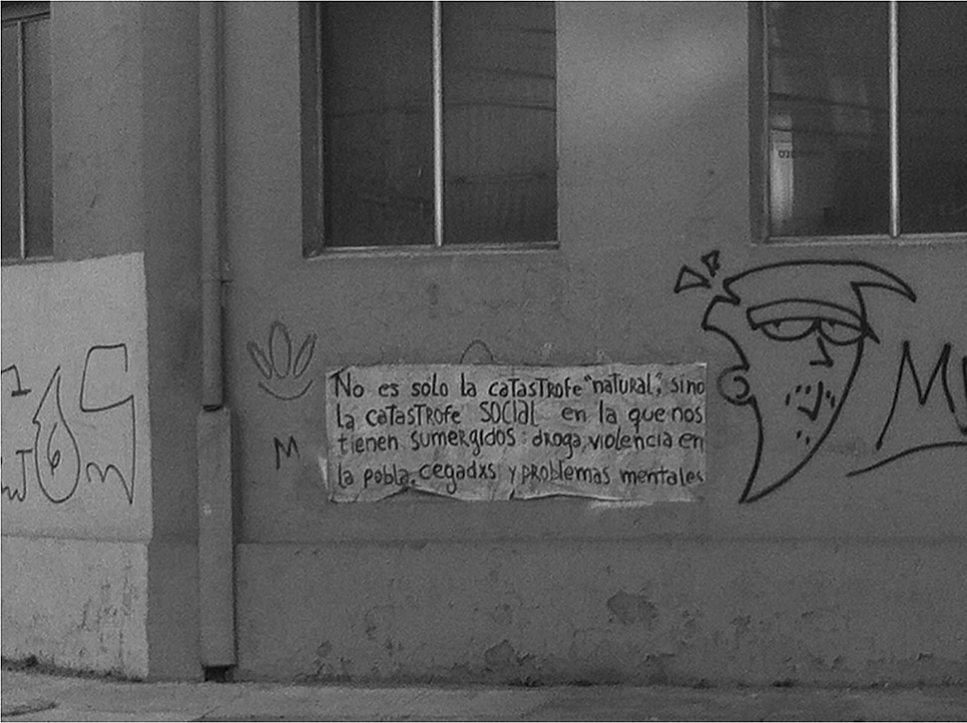
Valparaíso, Chile, street art near the harbor analyzing the meaning of disaster. The wall message says: “It is not just the ‘natural’ catastrophe, but a SOCIAL one that we have been submerged in.” Photograph by Meriläinen, 2015

Upon returning to Finland, the first author kept revisiting the data/creata, analyzing them, and making sense of them. Yet the creata were certainly not pure and untouched by theory, but creata often quite visibly contained its own analysis. First, there were the theoretical preconceptions of the researcher, layered in, through curation and construction of the exhibits that count as creata. Second, other research participants had theorized the disaster, and disasters more generally. For example, Fig. 3 shows street art in Valparaíso analyzing a catastrophe, stating that the catastrophe being experienced is not merely “natural,” but also SOCIAL.

The creata were built between the space-time trajectories of the researcher and other research participants, tinted with theorizing. While the pictures, notes, and recordings were labelled, locked supposedly in time and place, the creata ebbed and flowed between the moment of the hazard (and its build up), the time when the creata were constructed, and the time when they were again evoked and revisited by the researcher.

## Discussion

In the previous section of this article we illustrated how the space-time trajectories (Massey 2005) of the researcher, research participants, and disaster became entangled in complex ways in creation of data/creata. In qualitative research, creata act as a bridge across space-time trajectories. The illustrations reveal various temporalities and spatialities at play in qualitative disaster research, highlighting that the temporality and spatiality of creata are more complex than what any date and place labels might suggest. Qualitative researchers ebb and flow between the particular (for example, realities in the field) and the general (realms of theorizing), and analysis of the illustrations reveals that also the space-time trajectories are entangled on various levels of abstraction. The illustrations draw particular attention to how the methodology of fieldwork may end up reinforcing the hazard as an event (see Bankoff 2004), even if decentering the event through a focus on long-term construction of vulnerability and unequal disaster risk might provide more meaningful theorization (Hewitt 1983; Gaillard 2019).

The first illustration showed how a hazard, a fire, unraveled unexpectedly. The buildup of the conditions for the hazard (for example, lightweight construction material) and the disaster (for example, fire impacting particularly low-income and informal neighborhoods) was in no way sudden (see, for example, Kelman 2018). While “vulnerability” can draw attention to these structural features and the long-term buildup of disasters, it alone does not sufficiently address or depict how the event labeled the “Great Fire of Valparaíso” unfolded and was perceived. The various research participants’ perspectives on the temporalities and spatialities of the disaster differed a year after the fire. For instance, the disaster was both past and ongoing, both ash in the sky kilometers away and homes being reconstructed in the affected hills. In many respects, the hazard could be interpreted as an event in place that allowed, or forced, space-time trajectories to entangle. The hazard formed a fixed point on space-time trajectories. Rather than understanding the event as a single moment on a linear temporal continuum that splits before from after (Aijazi 2014), we suggest viewing the hazard as temporally rooted in the events of 12 April 2014, where the “place” of the hazard acts as an “arena where negotiation is forced upon us” (Massey 2005, p. 154). Qualitative research is thought to fare particularly well in mapping social phenomena in real time (Silverman 2016), but the fieldwork illustrated in this article was not only taking place in the present, but was tracing the entanglements of space-time trajectories brought to the fore by the fire.

Disasters might bring space-time trajectories together, even if under unfair circumstances. As the second and third illustrations show, however, in conducting qualitative research, the research participants are not likely to make sense of the disaster as an isolated phenomenon. For instance, other disasters and everyday inequalities can shape people’s theorizing and accounts of the disaster, as well as their understandings of spatiality and temporality. Marginalized people, for instance, are likely to be exposed to various interwoven threats (Kelman et al. 2015) and may experience everyday risks, such as those related to livelihood, as more pressing than extraordinary events (Cannon 2008; Ruszczyk 2018). As research participants, the disaster-affected people might not come to center the hazard as an event. Meanwhile, dominant and formal political actors are likely to reinforce the notion of disaster governance phases, and through that process center the event (Anderson 2010; Aijazi 2014; Zebrowski 2019). Research participants are not likely to place their emphases regarding the phenomenon unwittingly either, as they are likely to care about its representation (Alvesson 2011).

In qualitative research, creata are not raw nuggets of reality to be extracted, but rather they can be seen to come to life between the experiences and lives of research participants and the documentation of those experiences (Savin-Baden and Howell Major 2013). As such, the creata are not only likely to reflect the various emphases on combinations of vulnerability and event, but also to be combined from various, often nonsynchronous, understandings of the temporalities and spatialities of the disaster.

The third illustration brings out also the role of the researcher in constructing creata across space-time trajectories. While both the research participants and researchers are parsing together meanings and theories of disaster, the “creata” are ultimately often constructed and curated by a positioned researcher who not only documents, but also embodies, the creata over time (Bendix-Petersen 2013). Creata may be a bridge across different space-time trajectories, but one constructed by and in relation to the researcher. A researcher’s positionality shapes creata and research in various ways, from access to the field to the ways a researcher constructs the world (Berger 2015). Being an “insider” is likely to come with a nuanced understanding of the context, even if researchers are often unlikely to entirely share the positionality of the most marginalized research participants (Mayorga-Gallo and Hordge-Freeman 2017) such as those most vulnerable to disasters. However, a more burning concern is that dominant “outsider” research perspectives are imposed upon places beyond their origin, be it within disaster studies (Gaillard and Gomez 2015) or social sciences more broadly (Massey 2004). The involvement of an “outsider” researcher in the aftermath of a disaster, as is the case in the illustrations, is likely to reinforce the centeredness of the hazard in construction of data/creata.

## Conclusion

We agree with other authors that disaster studies would benefit from shifting and diversifying research practices, epistemologies, and methodologies (Gaillard 2019; Fiddian-Qasmiyeh 2019). Here we have focused on the methodological realm, drawing attention to temporal and spatial sensitivity in qualitative disaster research. Temporality and spatiality are central, yet often implicit, concepts present in disaster studies. The article’s aim is to interrogate how researchers’ sensitivity to temporality and spatiality challenges the conventional notions and practices of “data” in qualitative disaster studies.

Disaster studies deal with the unfolding of complex and devastating phenomena in time and space. Although the unequal consequences of disasters, for people and places, tend to reflect the inequalities of long-term sociospatial configurations (Oliver-Smith 1979; Nygren 2018), hazards can develop in temporally and spatially unexpected ways. While it is more meaningful to study and theorize disasters in ways that decenter the event (Hewitt 1983; Mika 2019), as this article has illustrated, inductive (Lindell 2013) post-hazard research (Norris 2006) conducted by an “outsider” is likely to methodologically center the disaster. The eventness seeps into the creata on various levels of abstraction (from particularities of the field experience to generalities of theorizing) and through the entanglement of various space-time trajectories (researcher, research participants, and disaster). A hazard is likely to form a fixed point on space-time trajectories, and the affected place is likely to allow, even force, their entanglements to be negotiated (see Massey 2005).

While our contribution here is to disaster studies, we believe the field provides an appropriate illustration of the issues surrounding temporality and spatiality of creata in conducting qualitative research amidst the wider devastation that has been attributed to the “Anthropocene” (Steffen et al. 2007; Clark 2014). It is not meaningful to theorize Anthropocene as a set of events since both the makings of the phenomenon and the potential interventions call for addressing structural vulnerabilities. Yet, similarly as with disaster studies, methodology might end up centering the events and exceptions. The Anthropocene might come to be studied through its shifts, changes, turns, collapses, and migrations. Even if qualitative research might not (wish to) steer away from “events” and even spectacles, temporal and spatial sensitivity is required to realize how various temporalities and spatialities are constructed into the creata. These temporalities and spatialities have a bearing on how knowledge is produced, but they also influence what kind of politics can be envisioned.

## Footnotes

1 The appropriateness of the Anthropocene term is being contested and debated both within the geological and the socio-environmental (for example, Moore 2017) research communities. However, we find it to be an umbrella term that usefully brings together various concerns for the stability of the Earth system and the conditions for human life.

2 Temporality and spatiality refer to how time (see Ialenti 2017) and space (Soja 1985), respectively, are understood and organized socially.

3 A notion adopted by Bendix-Petersen (2013) to highlight that “data” is *created* or constructed.

4 Research participants’ names have been anonymized.
